# Supplementary material for: The chloroalkaloid (−)-acutumine is biosynthesized via a Fe(II)- and 2-oxoglutarate-dependent halogenase in Menispermaceae plants
Source: Nat Commun. 2020 Apr 20;11:1867. doi: 10.1038/s41467-020-15777-w (PMC7170919; doi:10.1038/s41467-020-15777-w)
Supplement: Supplementary file 1 — Supplementary Information [file 41467_2020_15777_MOESM1_ESM.pdf]

Supplementary information for

The chloroalkaloid (–)-acutumine is biosynthesized via a Fe(II)- and 2-oxoglutarate-dependent halogenase in Menispermaceae plants

Kim et al.

*Corresponding author email: wengj@wi.mit.edu

Supplementary Figures

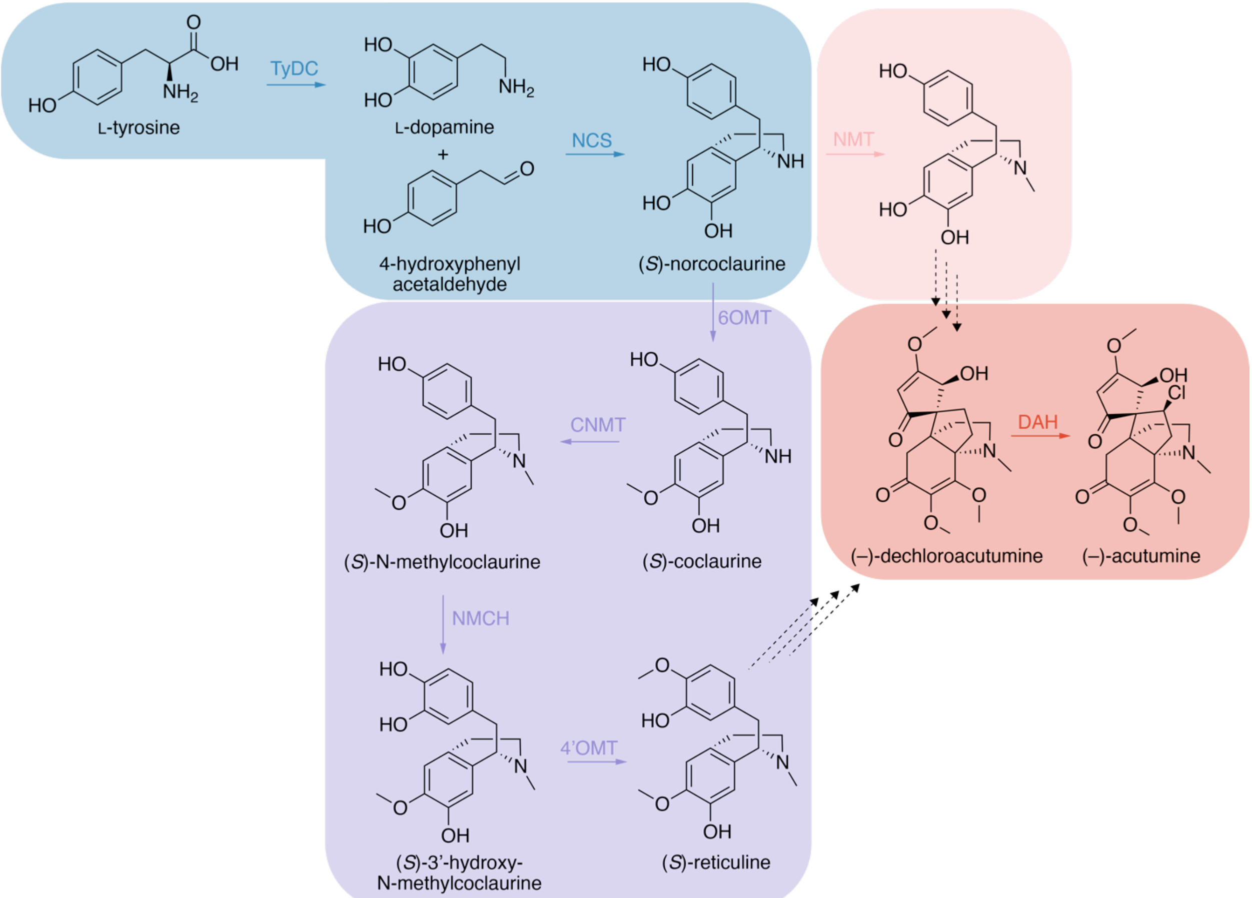

Supplementary Figure 1 | The proposed (–)-acutumine biosynthetic pathway. The labeled candidate enzymes were predicted from the *M. canadense* transcriptome based on homology search. Enzymes with high expression in *M. canadense* root tissue and annotation to BIA biosynthesis were chosen as representative candidates, but remain functionally uncharacterized. Abbreviations: TyDC, tyrosine decarboxylase; NCS, norcoclaurine synthase; NMT, norcoclaurine *N*-methyltransferase; 6OMT, (*RS*)-norcoclaurine 6-*O*-methyltransferase; CNMT, (*S*)-coclaurine N-methyltransferase; NMCH, (*S*)-*N*-methylcoclaurine-3-hydroxylase; 4′OMT, (*S*)-3′-hydroxy-*N*-methylcoclaurine 4′-*O*-methyltransferase; DAH, dechloroacutumine halogenase. The coloring scheme of the annotated enzymes is identical to that shown in Figure 1c.

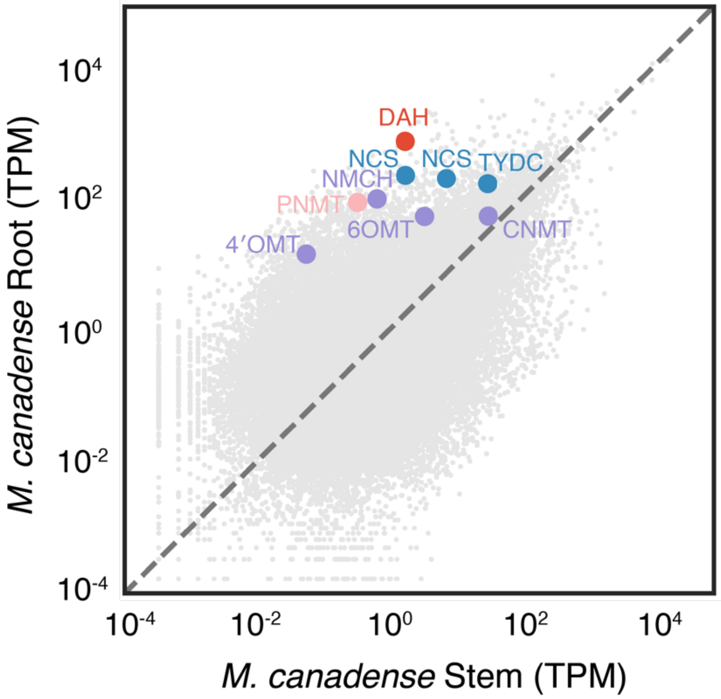

Supplementary Figure 2 | Differential expression analysis of *M. canadense* root vs. stem transcriptomes. Transcripts are quantified based on TPM values derived from the *M. canadense* root (6 biological replicates) vs. stem (3 biological replicates) transcriptomes. Candidate enzymes possibly involved in (–)-acutumine biosynthesis are denoted by colored dots. The coloring scheme of the predicted candidate enzymes is identical to that shown in Figure 1c. Abbreviations: NCS, norcoclaurine synthase; TYDC, tyrosine decarboxylase; PNMT, pavine N-methyltransferase; 6OMT, (*RS*)-norcoclaurine 6-*O*-methyltransferase; CNMT, (*S*)-coclaurine *N*-methyltransferase; NMCH, (*S*)-*N*-methylcoclaurine-3-hydroxylase; 4′OMT, (*S*)-3′-hydroxy-N-methylcoclaurine 4′-*O*-methyltransferase.

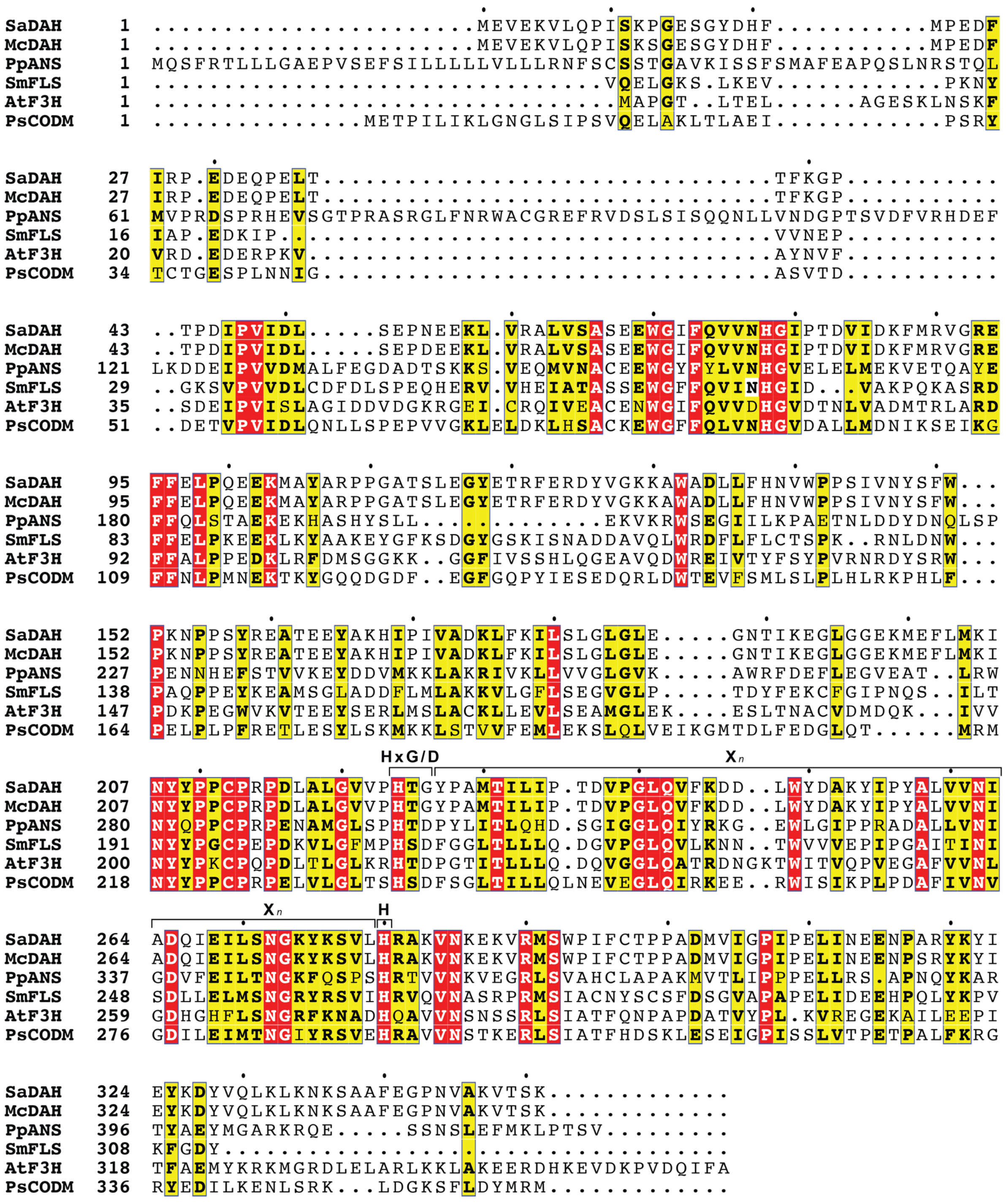

Supplementary Figure 3 | Multiple sequence alignment of DAH with other select plant 2ODD sequences. Included are *S. acutum* DAH, *M. canadense* DAH, *P. patens* ANS, *S. moellendorffii* FLS, *A. thaliana* F3H, and *P. somniferum* CODM. The multiple sequence alignment was built using MUSCLE[1] and visualized using the Flashy color scheme in ESPript 3[2]. The iron(II)-coordinating facial triad residues and the D226G substitution (HxG/DX$_n$H) are annotated.

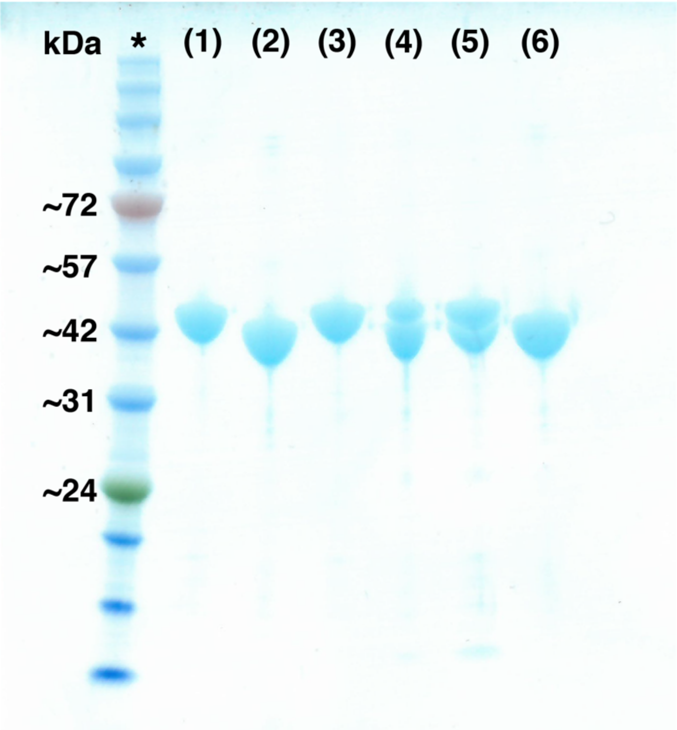

Supplementary Figure 4 | Protein expression and purity of recombinant SaDAH, McDAH, and SaDAH-G226D variant. The lanes in the gel correspond to the following: (1) SaDAH with 8xHis-tag, (2) SaDAH without tag, (3) McDAH with 8x His-tag, (4) McDAH without tag, (5) SaDAH-G226D with 8xHis-tag, (6) SaDAH-G226D without tag. The asterisk indicates the lane for BlueStain^{TM} protein ladder (Goldbio).

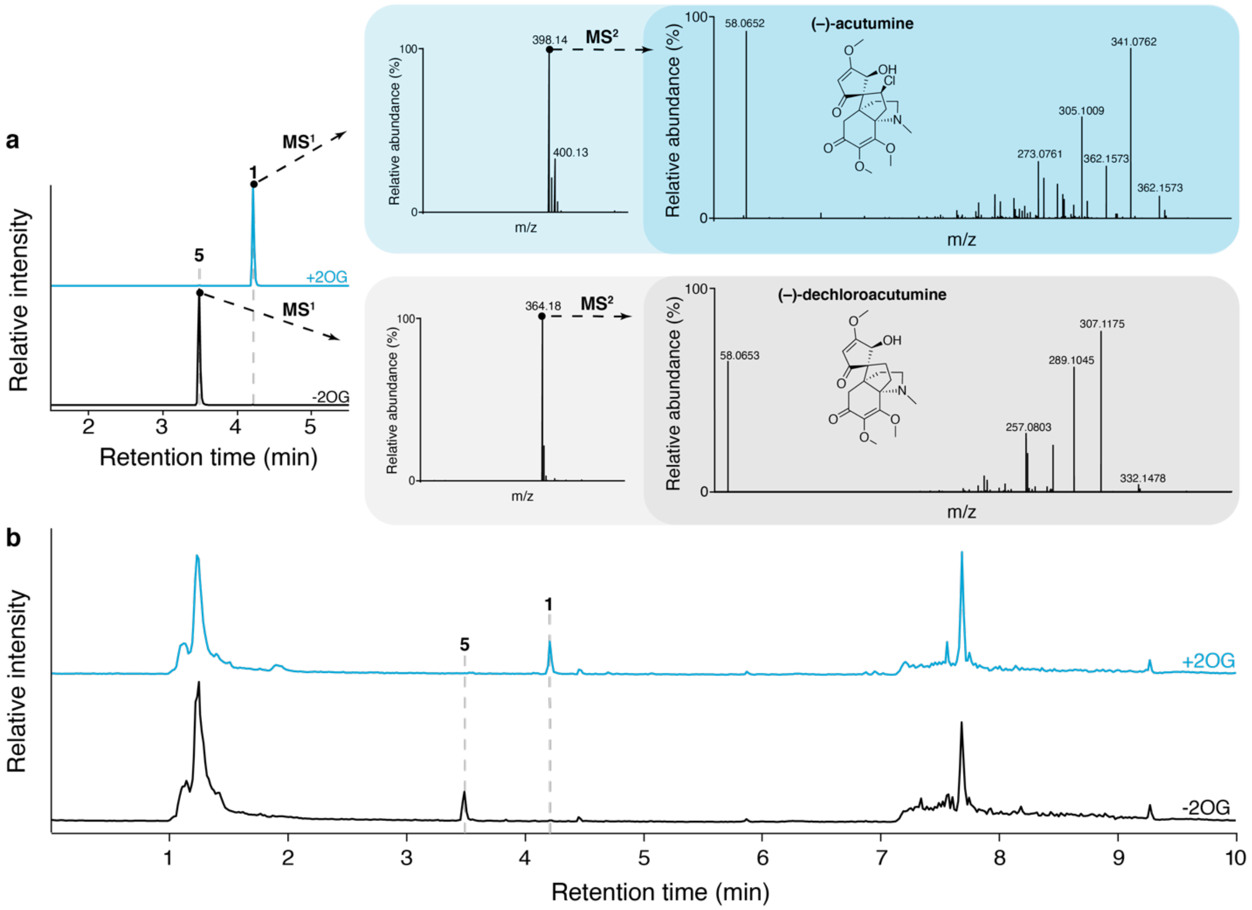

Supplementary Figure 5 | MS^1 and MS^2 fragmentation spectra of (–)-dechloroacutumine and (–)-acutumine in SaDAH enzyme assay. (**a**) LC-HRAM-MS analysis of the SaDAH *in vitro* enzyme assay identified a product at retention time of 4.21 min (**1**), which has a MS^1 spectrum corresponding to the $[M+H]^+$ value of (–)-acutumine. The presence of a chlorine atom is indicated by the $^{35}Cl/^{37}Cl$ isotope abundance ratio of 3.0. The MS^2 analysis of the 398.14 *m/z* ion is shown in the top-right corner, which matches that of the fragmentation pattern of (–)-acutumine standard purchased from BOC sciences. The MS^1 and MS^2 spectra for (–)-dechloroacutumine are also shown in the bottom-right corner with a retention time of 3.49 min. Both MS data were obtained in positive ionization mode with a full-scan range of 100-600 *m/z* and top five data-dependent MS/MS scans. (**b**) TIC chromatogram of the SaDAH *in vitro* enzyme assay. The peaks corresponding to (–)-acutumine and (–)-dechloroacutumine are indicated as **1** and **5**, respectively. There are no side products observed in this assay.

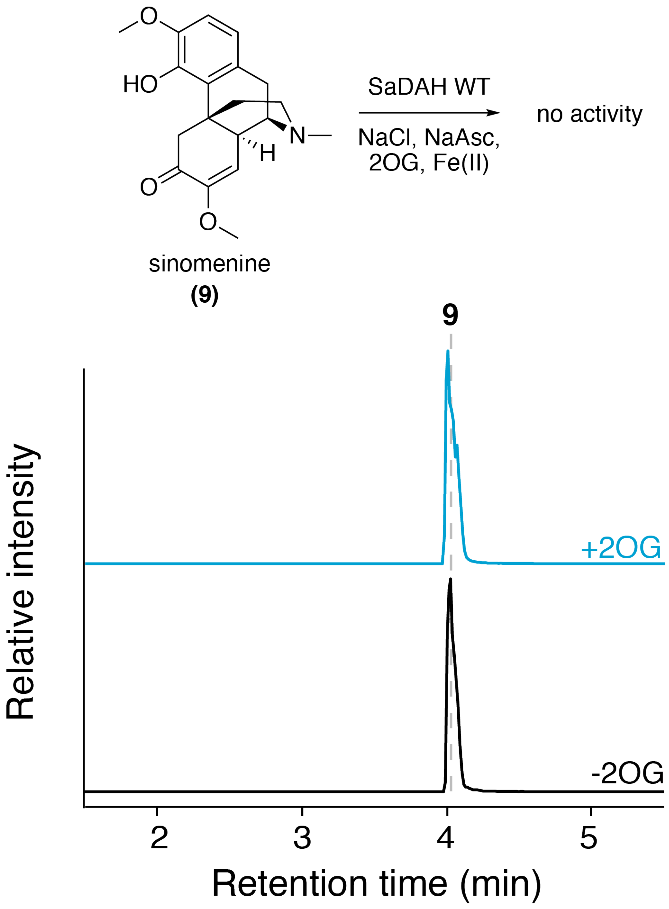

Supplementary Figure 6 | LC-MS analysis of SaDAH halogenase activity against sinomenine. SaDAH chlorinase activity was tested against sinomenine, a benzylisoquinoline alkaloid structurally related to (–)-dechloroacutumine. No chlorinated product was identified in the +2OG assay (blue) compared to the -2OG negative control assay (black) at the presence of 200 μM of sinomenine. The displayed TIC mass window is 300-400 *m/z*.

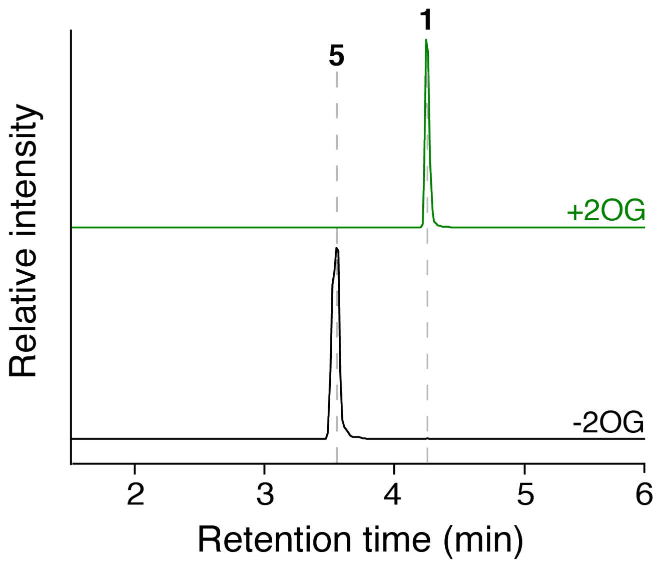

Supplementary Figure 7 | LC-MS analysis of in vitro enzyme activity of McDAH against (–)-dechloroacutumine. McDAH assay yielded a product **1** at the retention time of 4.21 min, which is identical to the result of the SaDAH *in vitro* activity assay. The MS^1 spectrum of the product obtained under positive ion mode corresponds to the $[M+H]^+$ value of (–)-acutumine, and the presence of a chlorine atom is indicated by the $^{35}Cl/^{37}Cl$ isotope abundance ratio of 3.0. The retention time and MS^2 analysis for 398.14 *m/z* matches those of the (–)-acutumine standard.

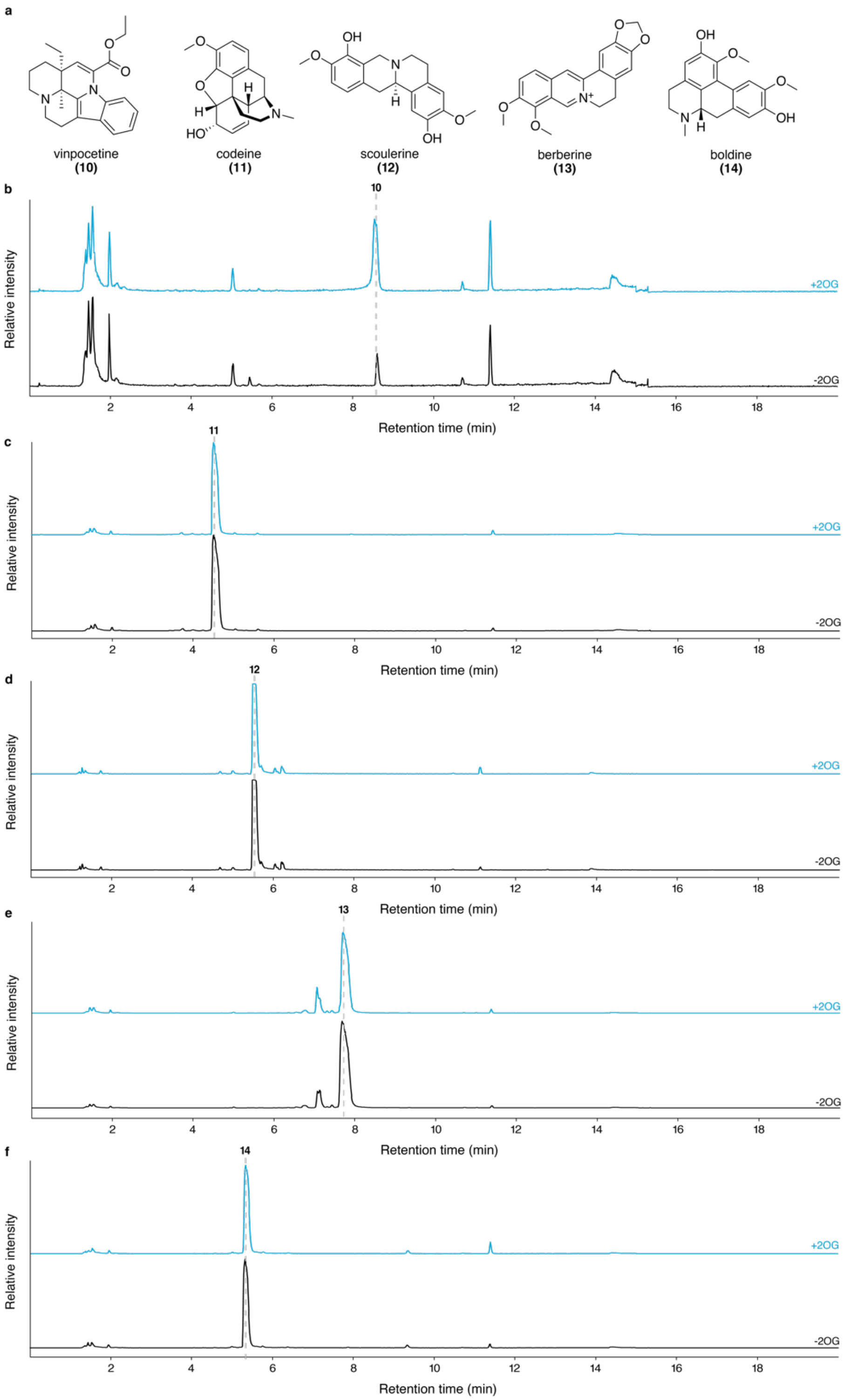

Supplementary Figure 8 | TIC chromatograms of SaDAH substrate promiscuity tests. SaDAH chlorinase activity was tested on structurally related alkaloids shown in (**a**). No chlorinated product was identified in the +2OG assay (blue) compared to the -2OG negative control assay (black) at the presence of 200 μM of (**b**) vinpocetine, (**c**) codeine, (**d**) scoulerine, (**e**) berberine, and (**f**) boldine. The displayed TIC mass window is 300-400 m/z.

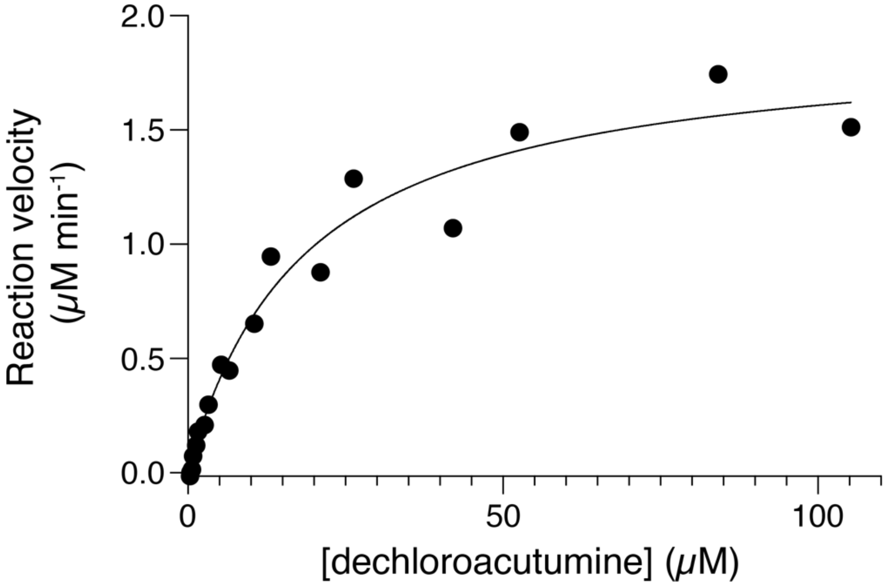

k_{cat} (min^{-1})	K_M (μM)	k_{cat}/K_M (min^{-1} μM^{-1})
63.44 ± 8.75	18.35 ± 7.13	3.46 ± 1.42

Supplementary Figure 9 | Steady-state kinetic analysis of SaDAH against (–)-dechloroacutumine as the substrate. The k_{cat} and K_M values and their associated errors were inferred from nonlinear curve fitting to the Michaelis-Menten equation in GraphPad Prism (v. 7.0). The error of the k_{cat}/K_M value is obtained by propagation from the individual kinetic terms.

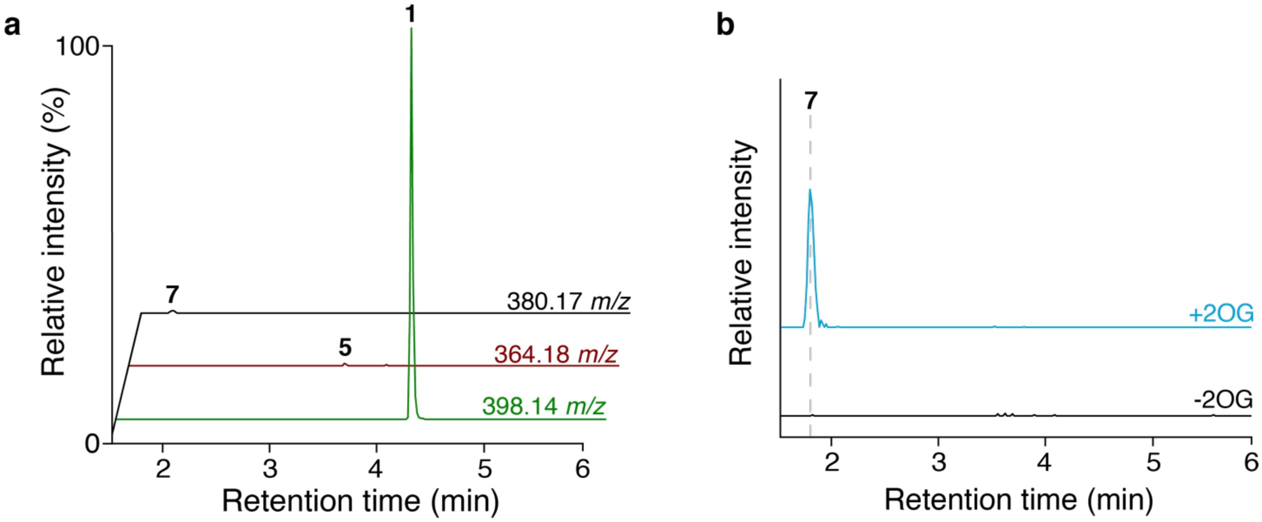

Supplementary Figure 10 | Production of trace amounts of 11-hydroxy-dechloroacutumine in SaDAH assay. (**a**) LC-MS chromatogram of SaDAH +2OG assay. XICs of **1** (398.14 *m/z*, green), **5** (364.18 *m/z*, red), and **7** (380.17 *m/z*, black) indicate the presence of the hydroxylated product (< 2% of the chlorinated product **1**). (**b**) LC-MS analysis of enzyme assays conducted with and without 2OG. XIC of **7** (380.17 *m/z*) indicates the formation of trace amount of the hydroxylated product **7**, when SaDAH is reacted with 1 at the presence of 2OG.The -2OG (black) and +2OG (blue) chromatograms are scaled to the highest peak intensity, $5.35{\times}10^5$ ion count.

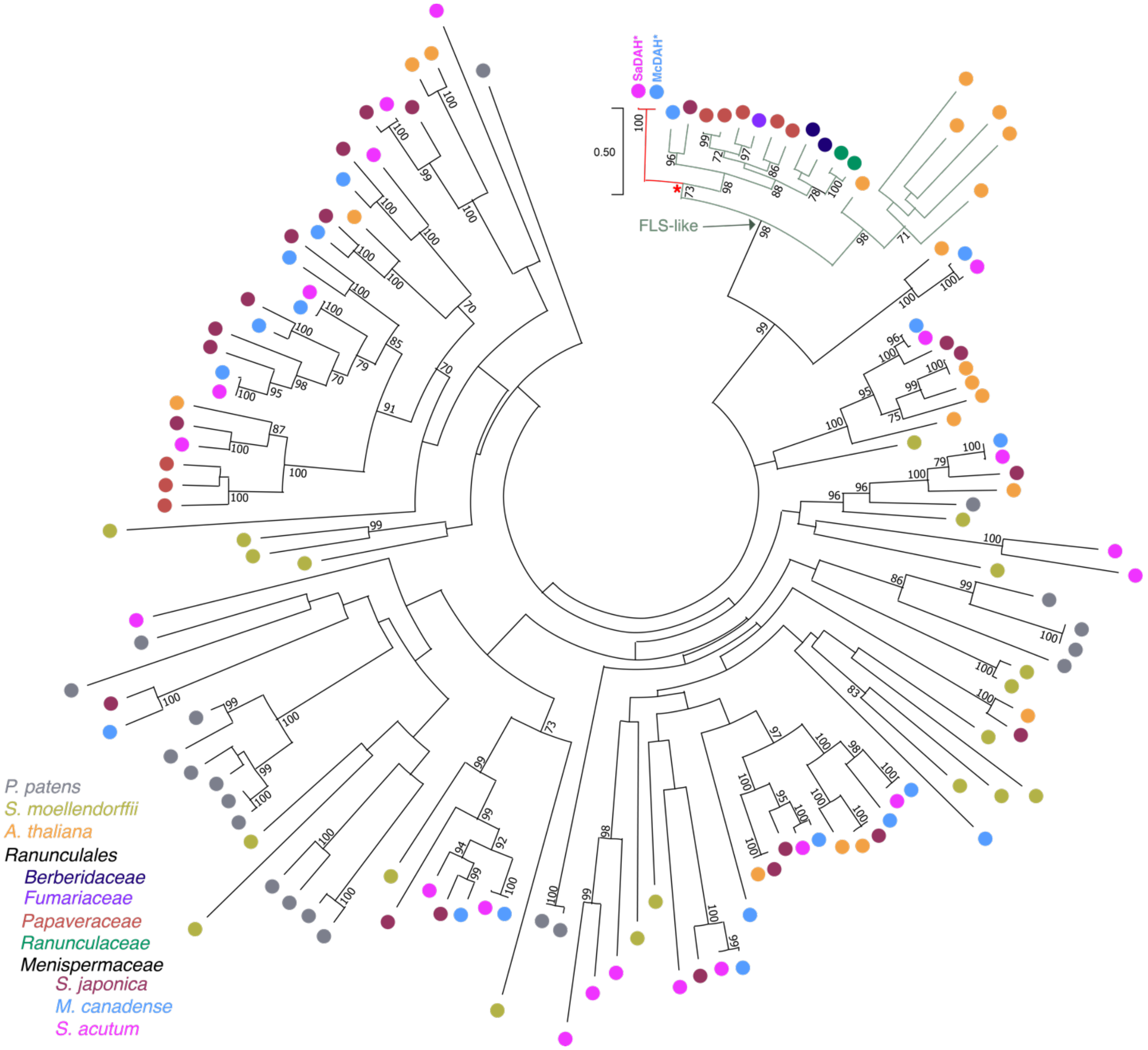

Supplementary Figure 11 | Extended phylogenetic tree analysis of DAH with other select plant 2ODD sequences. The phylogenetic tree of DAHs together with select 2ODD-family proteins is inferred using Maximum-likelihood method. Bootstrap statistics (200 replicates) greater than 70% are indicated at the tree nodes. The scale measures evolutionary distance in substitutions per amino acid. Asterisks denote the two orthologous DAHs identified in this study, along with the location of the proposed gene duplication event in red. The green branches indicate FLS-like sequences from Ranunculales plants and *A. thaliana*. The multiple sequence alignment used for building the phylogenetic tree is in Supplementary File 2. Abbreviation: FLS, flavonol synthase.

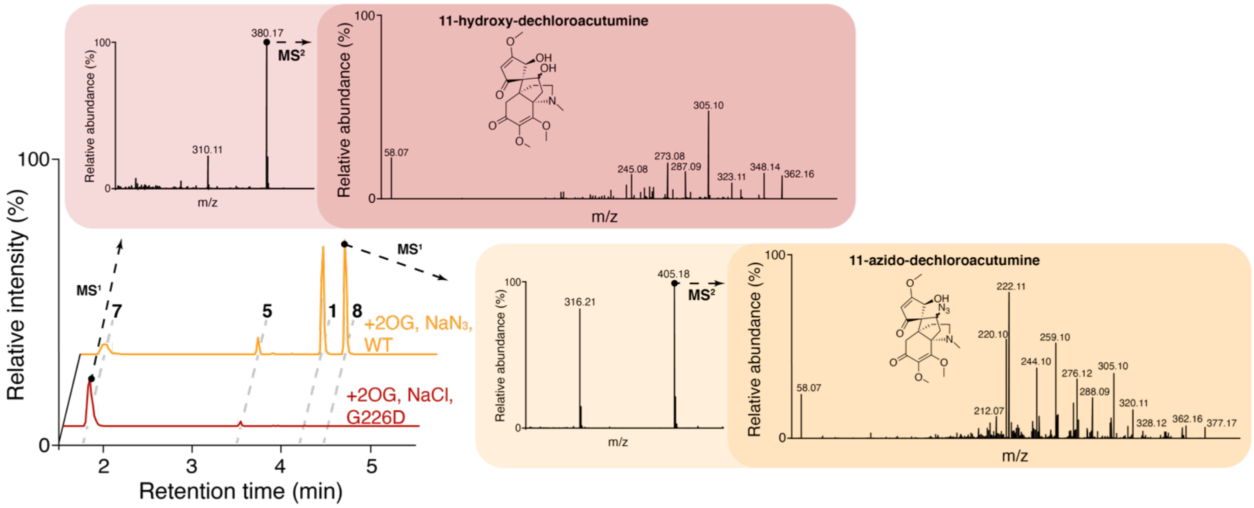

Supplementary Figure 12 | MS^1 and MS^2 fragmentation spectra of the major products from SaDAH-G226D (top) and SaDAH azidation reaction. LC-HRAM-MS analysis of the SaDAH-G226D *in vitro* enzyme assay identified a major product **7** at the retention time of 1.81 min, with an MS^1 spectrum corresponding to a hydroxylated (–)-dechloroacutumine (top). The MS^2 analysis of the 380.17 *m/z* ion is shown in the top panel. The SaDAH-WT assay with 1 mM NaN_3 yielded a major product **8** at the retention time of 4.46 min, with an MS^1 spectrum corresponding to a azidated (–)-dechloroacutumine (bottom). The MS^2 analysis of the 405.18 *m/z* ion is shown in the bottom panel, and exhibits ion fragments similar to (–)-acutumine with *m/z* values -N_2 fragments. The regio- and stereo-specificity of the -OH and -N_3 incorporations is unknown, but can be reasonably postulated to be installed at the C11 position of (–)-dechloroacutumine following the same stereochemistry as the -Cl group in (–)-acutumine. Since only a single -Cl product is observed in SaDAH-WT chlorination assay, it suggests that the enzyme is not capable of abstracting an alternate C-H bond. This postulation is consistent with the nature of substrate binding and C-H bond abstraction previously reported in other 2ODHs[4]. Moreover, the *m/z* transitions from 323.11->305.10 in **7** and 320.11->305.10 in **8** correspond to the loss of -OH and N•, respectively. This observation is consistent with the *m/z* transition from 341.08->305.10 in **1** that has been previously reported in a proposed loss of -Cl in MS^2 analysis of **1**[5] and leads us to support the regiochemical assignments of **7** and **8**. Both MS data were obtained in positive ionization mode with a full-scan range of 100-600 *m/z* and top five data-dependent MS/MS scans.

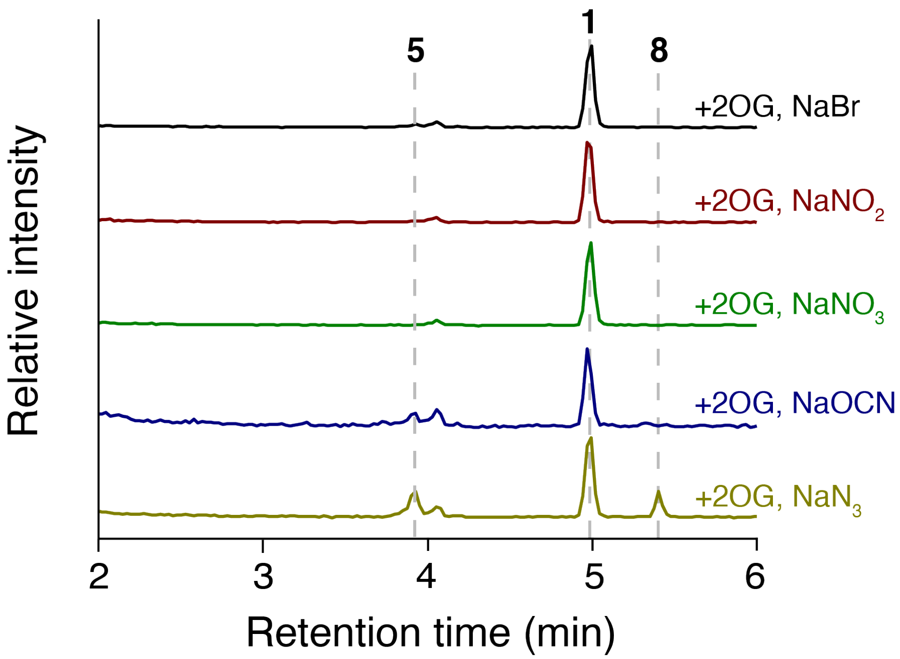

Supplementary Figure 13 | LC-MS analysis of SaDAH reactivity with alternative anions. The enzyme assay was carried out by replacing NaCl with each of the following salts: NaBr, $NaNO_2$, $NaNO_3$, NaOCN, and NaN_3 (1 mM each). Single ion monitoring (SIM) was used to scan the *m/z* values of the expected compounds: 364.20 (**5**), 398.1 (**1**), 405.17 (**8** and potential NaOCN assay product), 409.16 (potential $NaNO_2$ assay product), 425.16 (potential $NaNO_3$ assay product), and 442.08 (potential NaBr assay product) with scan width of 0.5 and collision energy of 20 V. The LC gradient was modified to achieve better resolution of the alternative product formation: 5% B for 0.5 min, a gradient of 5-60% B for 15 min, 95% B for 2 min and 5% B for 3 min, with a flow rate of 0.5 ml min^{-1}. A new peak was detected for NaN_3 assay corresponding to the expected azidation product (**8**, 405.17 *m/z*), which was further characterized using LC-HRAM-MS on Q-Exactive benchtop Orbitrap mass spectrometer (Thermo Fisher Scientific) (Supplementary Fig. 12).

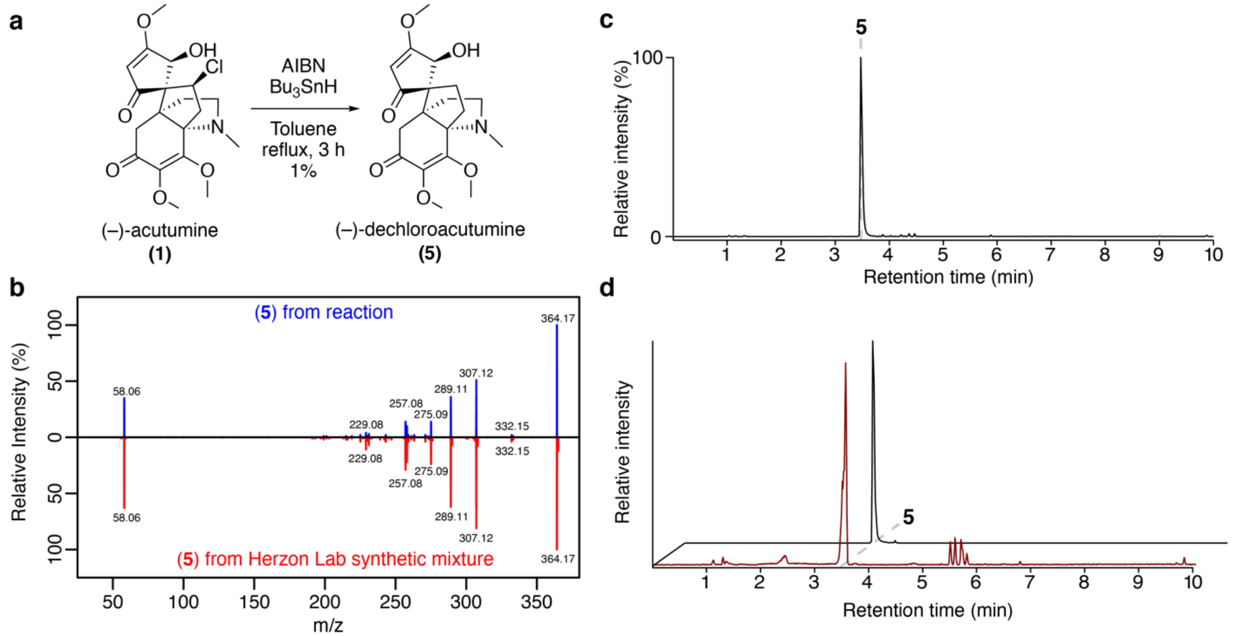

Supplementary Figure 14 | Organic synthesis of (–)-dechloroacutumine by dechlorination of (–)-acutumine. (**a**) A solution of **1** (6.0 mg, 0.015 mmol) in dry toluene at 23 °C was treated successively with $^{n}Bu_3SnH$ and recrystallized AIBN under N_2 atmosphere via procedure in Supplementary Methods. The crude reaction was further purified using prep-HPLC. This dechlorination reaction yielded an amorphous white solid compound of **5** (30 µg, 1%). (**b**) The MS/MS fragmentation pattern of **5** is in agreement with the spectral values by King, *et al*[6]. The spectral cosine similarity score of 0.7853 was calculated using the OrgMassSpec v0.4-4 package in R with *m/z* tolerance at 0.005 and baseline threshold at 34%. (**c**) TIC of the purified **5** shows no significant side-products from the dechlorination reaction and purification process. (**d**) XICs of **5** (364.17 *m/z*) for product from the dechlorination reaction (black) and the synthetic mixture from King, *et al*[6] (red).

Supplementary Tables

Supplementary Table 1 | Synthetic gene sequences reported in this study.

Gene	Sequence (5′ - 3′)
SaDAH	ATGGAAGTCGAGAAGGTACTCCAACCAATCAGCAAGCCAGGGGAATCGGGCTATGATCACTTCATGCCGGAGGACTTTATTCGTCCAGAAGACGAACAGCCAGAATTAACAACCTTTAAGGGCCCAACCCCGGACATTCCGGTTATTGACTTGTCGGAACCGAACGAAGAAAAGTTGGTTCGTGCCTTGGTATCAGCATCCGAGGAGTGGGGCATCTTTCAAGTTGTTAATCACGGGATTCCGACAGATGTTATTGACAAATTTATGCGGGTTGGGCGGGAGTTTTTCGAACTGCCGCAGGAGGAAAAGATGGCATATGCGCGGCCACCAGGGGCGACCTCACTGGAGGGCTACGAGACCCGTTTCGAACGGGATTATGTGGGCAAAAAGGCGTGGGCTGACCTCCTGTTTCACAACGTCTGGCCACCAAGCATTGTTAACTATTCCTTCTGGCCGAAAAATCCTCCTTCATACCGCGAAGCCACAGAAGAATATGCTAAACATATTCCGATTGTCGCGGATAAGCTTTTTAAAATCCTTAGTCTGGGTCTCGGCCTTGAGGGGAACACAATTAAAGAAGGTTTAGGGGGCGAGAAGATGGAATTTCTTATGAAAATCAATTATTACCCTCCTTGTCCGCGCCCTGATTTGGCTCTTGGTGTAGTCCCTCATACAGGCTATCCTGCAATGACAATTTTGATCCCTACAGATGTACCGGGCCTTCAAGTGTTCAAGGATGACCTGTGGTATGACGCCAAGTATATTCCATACGCCCTCGTGGTCAATATTGCTGATCAAATCGAGATTCTTAGCAATGGTAAGTACAAGAGTGTACTCCACCGCGCGAAAGTCAATAAAGAGAAGGTTCGCATGTCCTGGCCGATTTTTTGTACGCCTCCGGCAGATATGGTTATTGGCCCTATCCCTGAATTGATCAACGAAGAGAACCCGGCTCGGTACAAGTACATTGAATACAAAGATTATGTCCAGTTGAAACTTAAAAATAAGTCTGCAGCCTTCGAGGGCCCGAACGTAGCGAAAGTGACCTCAAAATAG
McDAH	ATGGAAGTTGAAAAAGTCCTCCAGCCGATTTCCAAGTCTGGTGAGTCGGGGTATGACCACTTCATGCCAGAGGATTTCATTCGTCCTGAAGATGAACAACCAGAATTGACCACATTCAAGGGCCCGACGCCTGATATCCCGGTGATTGATTTATCTGAACCGGACGAAGAGAAGCTTGTACGCGCGTTAGTATCAGCTTCCGAAGAATGGGGCATCTTCCAAGTCGTCAATCACGGTATTCCAACGGATGTAATCGACAAGTTCATGCGCGTTGGTCGCGAGTTCTTTGAGTTACCTCAAGAAGAAAAGATGGCTTACGCGCGCCCACCTGGGGCCACCTCTTTGGAGGGGTACGAAACGCGGTTTGAACGTGATTACGTAGGGAAAAAGGCTTGGGCAGACTTGTTGTTCCATAATGTGTGGCCACCAAGCATTGTAAATTACTCTTTCTGGCCTAAGAATCCACCGTCATATCGTGAAGCAACGGAAGAGTACGCGAAGCATATTCCGATTGTTGCAGACAAGTTATTCAAAATTCTGTCGTTGGGCTTAGGTCTTGAAGGCAATACCATTAAGGAGGGGTTAGGCGGTGAGAAAATGGAATTTTTGATGAAGATCAATTACTACCCGCCATGTCCTCGTCCTGATCTGGCATTGGGGGTGGTTCCTCATACTGGTTACCCTGCCATGACAATTCTGATTCCTACAGATGTACCTGGGCTCCAAGTCTTTAAGGATGACTTATGGTATGATGCTAAATATATCCCTTATGCGCTCGTGGTGAACATTGCGGATCAGATCGAGATCCTTAGTAATGGTAAGTATAAGTCCGTATTGCATCGGGCGAAGGTTAATAAAGAGAAAGTTCGGATGTCATGGCCTATCTTTTGCACGCCTCCAGCAGATATGGTGATCGGCCCGATCCCTGAACTGATTAACGAAGAAAACCCTTCCCGGTATAAGTACATTGAGTATAAAGATTACGTACAGCTGAAACTGAAGAACAAGTCGGCCGCTTTTGAGGGGCCGAACGTAGCGAAAGTCACATCTAAATAG

Supplementary Table 2 | cDNA sequences from *M. canadense de novo* transcriptome.

<table>
<tr><th>Name</th><th>BlastX annotation</th><th>Sequence (5′ - 3′)</th></tr>
<tr><td>DN7930_c0_g1_i2 (DAH)</td><td>sp|Q9ZWQ9|FLS_CITUN Flavonol synthase/flavanone 3-hydroxylase OS=Citrus unshiu OX=55188 GN=FLS PE=1 SV=1 E=3.32e-149</td><td>GATACACAACACCACATATCTTGATGCAAAGCTAACTCACTTA
AATGAAAAAAAAGTAAGTAATGTCTGTTGAATATTGAGTTATA
AAATCATTTTCCTATATTTTATTATCCATCCAGTCAAGCATGAC
CGGTGTGTTGTTTGCACAATCAACCATCAACCTGTGAAAGAA
CAGCTCTACCTCTACGTTCTGCTCTCTTAACTACCAAACTATA
GTCATAAAGAATATTGCCTTTAAATGCAGAGTAGAAATCGATC
AAAACCACACGTTCAGTCCAATCTTTGCTAAGTAATTCGAGAA
GGTTAAGTGATCAATGGAGGTAGAGAAGGTACTGCAACCCAT
CTCCAAGTCTGGTGAGTCAGGTTATGATCACTTCATGCCTGA
AGACTTCATCAGGCCTGAAGACGAGCAGCCTGAGCTCACCA
CTTTCAAGGGTCCAACCCCAGACATTCCGGTGATCGATCTAA
GCGAACCGGACGAGGAGAAGCTGGTGAGAGCTCTTGTGAGT
GCCAGTGAAGAGTGGGGGATCTTCCAAGTGGTGAATCATGG
CATTCCGACCGATGTGATCGACAAGTTTATGAGGGTTGGGAG
AGAGTTTTTTGAGTTACCACAAGAGGAGAAGATGGCCTACGC
TAGGCCTCCTGGTGCAACATCTTTGGAGGGCTATGAAACAAG
GTTTGAGAGAGATTATGTGGGCAAGAAGGCTTGGGCTGATCT
CTTGTTCCACAACGTTTGGCCTCCCTCCATTGTTAATTACAGC
TTCTGGCCCAAGAACCCTCCTTCTTACAGGGAGGCTACAGAA
GAGTATGCGAAGCACATACCAATTGTGGCAGATAAATTGTTC
AAGATTCTGTCTCTTGGGCTAGGGCTTGAAGGAAATACAATA
AAAGAGGGACTTGGAGGAGAAAAGATGGAGTTTCTGATGAAG
ATCAACTACTACCCACCATGCCCACGTCCTGATTTGGCTCTT
GGTGTGGTTCCACACACTGGCTACCCTGCCATGACCATTCTC
ATACCCACTGATGTGCCTGGTCTTCAGGTCTTCAAAGATGAT
CTTTGGTATGATGCCAAATACATTCCGTACGCTCTCGTGGTTA
ACATAGCTGATCAAATCGAGATTCTGAGTAACGGCAAGTACA
AGAGTGTGTTGCATAGGGCAAAGGTGAACAAGGAGAAGGTG
AGGATGTCGTGGCCAATATTCTGCACACCACCTGCAGATATG
GTTATTGGGCCAATCCCGGAGCTGATTAATGAAGAAAATCCG
TCAAGGTACAAGTATATTGAGTACAAAGATTATGTGCAACTCA
AGCTCAAAAACAAGAGTGCTGCTTTTGAGGGTCCTAATGTGG
CCAAGGTCACCTCAAAATGATCAAGCTAAAGGACTACTATGC
AAAAATCTGGTTTCTATCTATCCCTAATAAAGGACACTCATGA
TGTTTATGTCCATCTCTGGAGGAACTAGGACATGTAATGTTGT
ACCAATTTGTGTGTTTTGTGATCGCAGTGGCATGCTTGCTGTA
TCTTGTTTATGTATAAATAAGTTATATATATGAAGATCATGATG
TTTCATGGTAAAAAAAAAAAAAAAAAAAAAAA</td></tr>
<tr><td>DN7335_c0_g1_i2 (TYDC-like)</td><td>sp|P54769|TYDC2_PAPSO Tyrosine/DOPA decarboxylase 2 OS=Papaver somniferum OX=3469 GN=TYDC2 PE=2 SV=1 E=0.0</td><td>CGAGTTTGGTCAGTCCGCGCTTCTCTCCGCGACCCTCCAATC
TCCTATAAAAGAACCCCAACACCCTCTCCTCTCAACCACAACA
GAAACCCAAACTCAACACACGTTAGTTTCCTCTCCTCCTCATC
TACTCCTCTGCAACTTCAAACTCAACACATTTCATTTCTTTGTG
TTGTGTTTGTTGATCGTGGCTACGTTGTATATATATATATACATAT
ATAACATGGGCAGCCTTCACGCGGAAGAACTTGAGACCATCT
CGGTGTGTCCAGAGAACCCTCTGGACCCAGAGGAGTTCAGG
AGGCAAGGCCACAAGATCATCGACTTCCTAGCTGATTACTAC
CGAGACGTCGAGAAATACCCGGTTCGGAGCCAGGTTGAACC
GGGTTATCTCCGCAAACTTCTACCCGAATCCGCTCCAAACAA
CCCGGAATCACTCGAAACCATCCTCCAAGACGTGGAGACCA
CAATTGTCCCAGGCATCACACACTGGCAGAGCCCCAACTACT
ACGCTTACTTCCCTTCTAGCGGCTCAGTCGCTGGGTTCATGG
GAGAGATGTTGAGCACTGGTTTTAATGTCGTTGGGTTCAACT
GGATGTCGTCTCCGGCCGCTACCGAGCTCGAGAGCATCGTT
ATGGATTGGCTCGGCAAGATGCTCAAGTTACCCAAGTCATTT
CTGTTCTCCGGCAACGGCGGAGGTGTTCTACAAGGGACCAC
TTGTGAGGCCATTTTGTGCACTCTAACAGCCGCGAGAGAAAG
AATGTTGAACGTTGTGGGGAGAGAGAATATCGGGAAGCTTGT
TGTGTACGGGTCGGATCAGACCCACTGCGCGTTGCAAAAAG
CGGGTCAGATCGCCGGGATAAACCCGAATAACTTCCGACCC</td></tr>
</table>

		GTTGCTACAAACAAAGACACATCATTCGGGTTGTCGCCGGAA<br>GCGCTCCGGGCGGCGGTGGAAGCCGACGTGGCAGCCGGAC<br>TGATCCCGCTTTTTCTTTGTGCCACTGTCGGCACGACCTCGT<br>CAACGGCGGTTGACCCGATTGGACCGTTGTGCGAGGTGGCG<br>AAGGAGCACGGGATGTGGGTTCACGTGGACGCGGCGTACG<br>CCGGGAGCGCGTGTATCTGCCCGGAGTTCCGGCATTTCATC<br>GACGGAGTCGAGAACGCGGACTCTTTCAGCTTGAACGCTCA<br>CAAGTGGTTCTTCACGACTTTGGACTGCTGTTGTCTTTGGGTT<br>AAAGAGCCAAGCGCGCTGGTGAAAGCTCTCTCGACGAATCC<br>AGAGTACTTGAAGAACAAAGCGACGGAGTCAAAGCAAGTCGT<br>GGACTACAAAGACTGGCAAATCGCGCTGAGTCGGCGGTTCA<br>GGTCAATGAAACTGTGGCTCGTGCTTCGGAGCTACGGAGTG<br>GCGAATCTGAGGAACTTTTTGAGGAGCCATGTGAAGATGGCG<br>AAGCAGTTCCAAGGGCTTCTTGGTATGGACAAGAGGTTCGAG<br>GTGGTGGTCCCGAGGACGTTTGCTATGGTGTGCTTTAGGCT<br>GGTATTTCCGAGTGAGCGAGTTGAATCGGCGGAGGAGAACT<br>GTTTAGAGAGAGAAAACGAGTTGAACAGGAAGTTTTTGGAGT<br>CGATGAACTCGGAGGGAAAAGTGTACATGACTCACGCGGTG<br>GTGGGAGGAGTGTATATGGTTCGGTTCGCGGTTGGGGCCAC<br>GCTGACGGAGGAGCGCCACGTGAACTTCGCGTGGAAGGTG<br>GTGCAGGATCACGCGGACCGAATTCTTGCTGACTTTTGATGA<br>TTTTTTTTTTAATTAATGGAGGAGATGCAGAAAGAAAGCAGAG<br>AGCTGTTGGGTTGGTATCGATTTCTTGAATTTGGAATACATGT<br>ATGTGGCTTTTTTTTCTTCTTATTTTGTGCCTATATTGTAAAAC<br>CTTCTTGTCAAATTATATCAATTTTATTCAATAATTACTCTTCTT<br>AAAAAAAAAAAAAAAAAAAAAAAAAAAAAAAAGGGGGGGGGC<br>AGATCGCCGGGATCAACCCGAA
DN4289_c1_g1_i8 (NCS-like)	sp|Q4QTJ1|NCS2_PAPSO S-norcoclaurine synthase 2 OS=Papaver somniferum OX=3469 GN=NCS2 PE=1 SV=1 E=3.98e-43	AGTAGCCACAAATCAATATACTAAAAGAAATGTTCAACTCACA<br>ACAACTCATCCAAGTTGAATTGTCTATATAAGCCAACCCAAGT<br>ACTCAATCTGAATATATCTACAACCAGTCGATCCATTTGAGTC<br>TATTAAGAGATCAAGAAGTGAAGCAGAAGAAGAAGAAGATGA<br>TGAAGGAGCTCAAGCATGAATCGGAGGTACCTGCCTCTGCA<br>GACGATATATGGGAAGTCTACAGCTCTCCCGATTTGCCCAAG<br>CTTATCCTTAAATTGCTTCCAGGCGTGTTTGAAAAGATTAAAA<br>TCTTGGAAGGCGACGGAGGTGTTGGCTCTATTCTTCGAGTCA<br>TATTCCCACCAGGGTCAGTGCCGCTTACTTACAAGGAGAAGT<br>TCGTGACCATTAACAACAGGAGGCGATTGAAGGAGGTGAAG<br>CAAATTGAAGGAGGGTACCTTGATATGGGCTGCACATTCTTC<br>AGAAGCAGTGTTCATATTTTACCGAAAGGTCTCAATTCATGTG<br>TGATCGTGTCTAAGATTGAGTACGAAGTACCCAATAACGAGA<br>TAGCAAGACAAGTTAAACCTTATATATCTATTGGTTCACTAGT<br>GAATCTGGCGACGGCCATCACGAATTATGTTTTAAACGGGGG<br>GAGAAGAAACTGAAGCATGAATTGGAGGTTGCAGTGTGTGCA<br>GATGATGTTCAAGAAGTGTTGGAATTTGGCTGAAACTTAAACT<br>AGTTTGAATATAATTTATGCGTTTGATTGGTATTTAGAGTCCAA<br>ATTGTAATAGGATTAAGTCTTTTATAAGTGCAGTTCTATAAATT<br>GAACCCACGGTTCATAGTTTATGTGTTCCGACTGTACTAGTTT<br>GAGCATTCTTAAATAAAAAATAGTTAAATTGATTAAAAAAAAAA<br>AAAAAAAAAAAAAAAAAAAAAAAAAGGGGGGGGGG
DN139_c0_g1_i2 (NCS-like)	sp|Q4QTJ2|NCS1_PAPSO S-norcoclaurine synthase 1 OS=Papaver somniferum OX=3469 GN=NCS1 PE=1 SV=1 E=3.55e-21	CTCCAGTTAGACAGCAACACAAGTAACCCTCATTCGACCCAT<br>CTATAAAATGCAATAGATCTTGACCCCAAGGTCAGCTTTCATT<br>TCCATTAAACACATCCTAGAATTGGCTTAATTTACAGAAGCAA<br>AGAGAATGCGAGGAGAGGTGTCCCTAGAGTTGGCCATAAGC<br>GCACCAGCCAAAGACGTCTGGGCTGCTATCGGCTCTCGCCA<br>AACGCCCAGCCTCATCCTTCGCTTGCTGTCCAGCATGTTCGA<br>GAAACTAGAAATCGTGAAAGGCGATGGAGGCCTTGGCTCCG<br>TTTTACATATCGTTCTTTCTCAAGGAGAGCCTCGGGAATGGAT<br>AGAGGAGATCGTGAAGATTGATGATGAGAAGCGCGAGAAGA<br>GGGTTCGGACGATCGAGGGAGGGTTTCTCGATCTGGGATTT<br>AGTTTCTACGAAGTGGTGTTCAACATCCTAGAGAAAGATGAG<br>TTTTCGAGCATTTACAAGTCGTCCATCGTTTTCGAAGCCGATG<br>ACGATAAGTTCCCGGCCGCTGCTTCTCTGGTCTCCGCCGCTT<br>CTGCGTGGGAGATTCCGCGTGCAATCGCAGACTATGTTGCG<br>CGCAACAAGGCCGCATAACATGTAACGTTGTACCTCGAGATA<br>TGCATCGTTTAAGCATCTGTTGGCTGTGTTTTTTCATATCGAG<br>TCTGTTTTGTTCATATATTTACTCTAATATCAATAAAAAAAAATTT

		ACTCTAATATCAATTAAAAAAAAACAGAATGTATGACACGATG<br>AATTGCTCGCATTTTAAAAATAAATGCATCTGAATAAGGATGA<br>TAAAGATAGTTTTTTTTAATATTAGCTCAAACATGATATACAGT<br>ATGTTAATAAGGAAATGTGATAAAATATTGAAAGATAAAATTA<br>GAAATGAATACAAATGGTATGAGTAGCCTCAATTGAAAATGAA<br>ATGATAGAAAATTGATTAAGATGGTTTTAATACATATAACGAA<br>GATCAGATGTACTAGTTAAAAAAAGTAATTTATTAACTATCTAG<br>GACAATAACAGAAGTAGAAAAAAATTTAAAATAATATTAACAG<br>AAATAATTAAAAAAAATATTATTATTTGTAATTTAAGAAAGATAA<br>TTCAT
DN9755_c0_g2_i5 (putative norcoclaurine N-methyltransferase candidate)	sp|C3SBW0|PNMT_THLFG Pavine N-methyltransferase OS=Thalictrum flavum subsp. glaucum OX=150095 PE=1 SV=1 E=6.92e-102	CTACAGGATCGGGGAGTCGTGGCAGACGCTTAAAGCACATC<br>GCTTTGTTTGCCATCAGCCTCTGCTGCAGACTCAACACTACA<br>CACAGCAGCCTCTGCATATATAATCTCTGTGTGTGAGCCTCA<br>ATTAATAGTCCCAATTCAGATTCATCTCAGCTTCTTTAATTTCT<br>TTCTTGCTTGCTTGCTTGCTTGATTAGTATAATTAGGTAGTGA<br>GGCTATGGATGCTAAGGAGGCTAAGAAGGAAAGTTGTGTTGC<br>AGAGCTGCTAGAGAGACCGGAGCTTGGGCTTGTACCAGACG<br>AAGAGATCAGAAAGCTCACGAGGACTCAGCTTGAAAAGCGTC<br>TCAGATGGGGTTACAAAGCCACCCATGAAGACCAACTCTCTC<br>ACCTTCTTCAATTCATCCAATGTCTACCATCTTTAAACATGGA<br>AAGTGAGGGTGATAAACCAAAATCTTGGTTGTATGAGACACC<br>AACTTCATTTCTTCAGCTTATCTATGGAGACACCATCAAAGAA<br>AGTAGCACATACTACAAGGATGAGTGGTCCACATTGGAAGAG<br>GCTATGACACACATCTTGGATTTGTGCTGTGAGAGGGCAAAG<br>ATACAAGAGGGCCAAAGAATTCTTGATCTTGGGTGTGGTTAT<br>GGAGCACTCACTGTACATGTTGCAAACAAGTACAAGAGTTGC<br>AGTGTCACAGGTGTTACTACCTCAATCTCTCAAAAACAGTACA<br>TCACGGAACAGTGCAAAAAACTCAATTTATCTAATGTTGAGGT<br>GATATTAGAGGATGTAGCAACAATGAAAATGGAGACGACATT<br>TGATCGAATATTTGCTCTGGGAATATTTGAGCATATGAATGAC<br>TACAAACTATTTCTTGGAAGAATTTCAAAATGGATGAAGCAAG<br>ATGGTCTTCTCTTTGTGGAATATTTGTGCAACAAGACCTTTGC<br>ATACCAAAATAAGCCAGTTGATGACGGTGATGATTGGTACAA<br>TGAGTATGTTTTCCCATCTGGTGG
DN3510_c0_g4_i1 (CNMT-like)	sp|Q5C9L6|CNMT_THLFG (S)-coclaurine N-methyltransferase OS=Thalictrum flavum subsp. glaucum OX=150095 PE=1 SV=1 E=0.0	TAGAGAAGTAAAAGGGTGGAACTTAGAAGCGCAGAATCGAAA<br>AACTTAAAATAGAGTGACACAATATCTACTACAAAATACACAC<br>ACTTTATTGTGGTATGTTGGATGTGCATCGATATATGTATTATT<br>ACCAAAGAAAAGTCGGCCGAAGCTCTTGCCTCTCTTGGGCGT<br>GTTTCACGTGAGCATGGACGACAAAACTCGCCTATTCTAGCG<br>TGCGTCACGTGCAGGGCGGTTGCTATCGAAGATGTTAGACTT<br>TCAAAGGCCTACCTACTAAGAATTACATAGTCCTGGGACTAC<br>ACCATCACTTTATATATATTACATTAGAAACCAACCGTTGAAA<br>GCCTAATTGGTTGCTTGGAACTGATCACTTGAGTCTCTGTTTC<br>TTGTATTATTGGTGTTTGGTGGTTTAATGGCTGTGGGATCAG<br>GAGATATGGAAGATAAGAAGGCAAGAGTGGCGGAGCTGCTG<br>AAGAAGCTAGAGCTGGGTCTGGTTCCGTATGATGAGATCAGA<br>CGGCTGATAAAGGTGGAGCTGGAGAGGCGCCTTCGATGGGG<br>TTACAAGCCAACTTATGAACAGCAAACTGCCGATGTCGTCAA<br>TTTCGCTCGTTCCCTACGTAAGATGAGCATTGCAACAGAGAT<br>CGATACATTGGACTCCCAAATGTACGAGGTGCCGATCTCATT<br>TTTAAAGCTTATGTTCGGAAACACAATCAAAGGAAGTTGCTGT<br>TACTTCAAAGATGACACTGTGACATTAGATGAGGCTGAGATA<br>GCAATGTTGGATTTGTACTGCGAGAGGTCACAGATCAAAGAT<br>GGTCAGCGAGTGCTCGATCTCGGGTGCGGCCAAGGTGCTCT<br>CACCATGCACGTCGCTCGCAAGTTTCGCAACTGTCGGGTCAC<br>AGGAGTCACCAACTCTGTGTCTCAGAAAGAGTTCATTGAAGA<br>GCAATGCAAGATAAACAACTTGCCGAATGTGGAGATCGTACT<br>AGCAGACATAACCACGCACAAAATGGATGATAGATTCGATCG<br>GATATTAGTTATTGAATTGTTTGAGCACATGAAGAACTATGAG<br>CTGCTTCTTAGGAAGATATCAGAATGGATGACACCAGATGGG<br>CTTCTTTTCATTGAGCATATTTGCCACAAAACTTTTCCCTATCA<br>CTATGAGCCTCTTGATGAAGATGACTGGTTCACAGAATACAT<br>CTTTCCGGCTGGGACTATGATCATACCATCAGCCAATTTTTTT<br>CTATATTTTCAGGATGATGTTTCGGTTGTGAACCATTGGACGC<br>TAAGCGGAAGGCATTATTCGCGTACCCATGAAGCGTGGCTGA<br>AGAACATTGATGCCAATGAAGATGCAGTGAAAGCAATAATGG

		AATCCTTCACAGGCAGCGAGGAGGCTGCGGTGAAGCTGATG<br>AACTACTGGAGAGGATTCAACTTATCTGGGATGGAGCTCTAC<br>AAGTACAAAAATGGTGAAGAATGGATGGCATCTCATGTCCTC<br>TTCAAGAAGAAATGATGCAACTCTGCAATTCATTATTTTTCTTG<br>GACAATGAGATCTTAAAACTATTCACCAATCTACAAAATAAATT<br>GTCACTCCATCGTCCATTTGAAACTAATTTGTTATAGTTGGGA<br>TTCAATAAAAAGCTGATGTTTTTGATGGACACTACCTACAAAA<br>TTCTCATCCCGTTCTACCCCACCCGATATTGTCTCGGCTACC<br>AAGGTTTTGTTCCCTCTCAGGGAAAAAAAAACGTCAATAGTGA<br>TTGAAAGGGATAGTACAATATAGCTCAATCTTGCTTCTATCAC<br>ACGTCTGATGTGAGAGACTATTACAACCTTACTTTCTAGTGCT<br>AGAACTTCTTCATTGGGTTCTTTTAGGATCATTTGCTACAGCT<br>ACTTTACTCCAGTTGTCCCTAGTGCTTCATCTGTGCTAAAAGA<br>TCTAGTATTTCAACATCATAACAGTACTATTCTATCAACACCAC<br>TTTATATATAAACGCGA
DN14058_c0_g1_i3 (6OMT-like)	sp|Q6WUC1|6OMT_PAPSO (RS)-norcoclaurine 6-O-methyltransferase OS=Papaver somniferum OX=3469 GN=6OMT PE=1 SV=1 E=2.78e-106	GAGAGAGCGAGCGAGAAAATGGAGGAAGACATGAAGGCTCA<br>AGCGCAGGTGTGGAGATACGTGTACGGCTTCGCCGAGTCAC<br>TGACTCTCAAATGCGCGATTCAACTCAGTATCCCCGACATCC<br>TCCACCAGCACGGCCGCCCCATGACTCTCTCCGAGTTAGCT<br>GACTCACTGCCCCTCCCCACCGTGAACCAGGACCGATTGTTC<br>CGAATCATGCGCTACCTAGTCCACATGGGACTCTTCGACCTA<br>GTCGACTCAGACAAAAAATACGCTCTCAACCCCGCTTCAAAC<br>CTCCTCATCAAAGGCCAAGACAAGAGCCTCGCCTCCTTTGCT<br>CTCCTCCAGTACTACGAGATGGACGCGTGGCACCACCTCAG<br>CGCGGCGGTGGAAGGCGGCGTTACGCCGTGGGAGAAGTGC<br>CACGGCGTGGACTACCGAGAGTACTTCGCGAAGGACTCGGT<br>AGCGAACCAGCTGCTGAGCGACGCCATGACGAGTCACACGA<br>GCATGGTGACAGAGGCGCTCGTGGAGGGATGTAAGAAGGCG<br>AAAGTGCTCGACGGGGTCGGCTCGTTCGTCGATGTCGGCGG<br>CAGCACCGGCGTCGCCGCTCGCGCCATCGCTAAAGCTTTTC<br>CGGGAGTCAAATGCGCGGTGTTTGATCTTCCTCACGTGATCG<br>CCACCGCGCCGGAGTGCGCCGAGGTGGAGCGGATCGAAGG<br>GGACATGTTCGTTTCCGTGCCTGAGACCGATGTGGTCTTCAT<br>GAAGTCTGTGTTGCACGACTGGGGAGATGAGGACTGTGTGA<br>AGATTCTAAAGAAGTGCAAAGAAGCGATTGGAGGGAGAAAAG<br>GGGGGAAAGTGGTGATACTGGACATAGTGTTGGATGCAGAG<br>TCGAGTTATGAGTTTAAGGGAGCGAGGTTGGGGATGGAGAT<br>GGACATGTTGGTGACGGTGGGAGGGAAGGAGAGGAGCGAG<br>GAGGAGTGGCAGAAGCTGTTCAAAGCTGCGGGTTACAGTCG<br>CTACAAGATCACGCCCATCGTCGCCATTGAATCCATCATCGA<br>ACTCTTCCCTTAAACAAAGATCAGTTTAGATGAGATGAGATGA<br>GATTGTTTGTTTTGTTTTCTTGACTTGTTTATGTGACGACAAAG<br>GCAGATCAATCAAGAATAGCTCATTGATAGAGAAATGCTGTTT<br>GATTTAATAAAAATCTGGAATTGGTGCGGT
DN29690_c0_g1_i4 (NMCH-like)	sp|Q9SP06|C80B3_PAPSO (S)-N-methylcoclaurine 3′-hydroxylase isozyme 1 (Fragment) OS=Papaver somniferum OX=3469 GN=CYP80B3 PE=1 SV=1 E=4.86e-61	CTCCACGACTGCATGAAGGAGACCTTGAGACTACACCCACCA<br>GTGACTTTCCTTCTGCCTCATCGAGCAACCGAGACGTGCCAA<br>GTGATGAACTACACGGTTCCGAAGGGGTCCCAGTTGATGGT<br>CAACACTTACGCGATTGGGAGAGATCCAAAGACATGGGACG<br>ACCCAAACTGTTTCACGCCAGAACGGTTTTTGAACTCAGAAG<br>TTGACTACCAAGGCAATGATTTTCATTACATACCGTTTGGAGC<br>TGGGAGGAGAATTTGCCCAGGATTGTCTCTGGCGTCTAGAGT<br>GGTGAGATTGATTTTGGCTTCTTTGATTATCAATTTTGACTGG<br>AGCCTGCCCAATGGGATGCACCCGAGTGAGCTAGACATGCA<br>GGACAAATTCGGATTGGCTCTTCTCAAGGACATTCCTCTCCT<br>GCTGGTGCCCAAAGTGAGAAGTACATGATTCGTGAATGAATG<br>GAGATGGGATTTGTCTCTTTGTTTGATTTCTTTCATATATGTTG<br>ATAAATTATGAGAACGTCGCTTGGTGTGGGTACGCCCTTTTAT<br>GTTGTTAAATTAAGAATATAAGTTGGGGTTATCTATAGAGGTT<br>AGCATCTTATTGCAGCGGCACCCTTGTCTTCAAGTGTGTACTT<br>GTATGTAAGACTTAATTTAAATACATATTTTTTTTTATCAAAAA<br>AAAAAAAAAAAAAAAAAAAAAAAAAAAAAAAAAAAAAAAAAA<br>AAA
DN48459_c0_g1_i2 (4′OMT-like)	sp|Q9LEL5|4OMT_COPJA 3′-hydroxy-N-	TTCATCATGAGAGACATCTGTCCATGACCATGCATGCCCATG<br>AAGACAGACAAGCAAAAAGAAAACTCTTTTGCTGTATAAATAG<br>AGTTGGCTAGCTCAAGTGTTGCAACATATAATTTCTTTGAGAT

	methyl-(S)-coclaurine 4′-O-methyltransferase OS=Coptis japonica OX=3442 PE=1 SV=1 E=2.35e-165	ATGGCACCAGAGATTGCTATTAATGAGATGATCTTGGACAAC<br>CATGACAAAGCAGAAGAAGCTTTAGTAGTGGATGTCCATGAC<br>CAAGCACACCTATGGAAGCTCATCTATGGCTTTGTGGACACC<br>TTAGTTGTTAGATCGGCAGTAGAGCTTGGAATCATGGACATC<br>ATCTACAACAACAAGAAGCCCATCTCACTCTTCGATCTCGCCT<br>CGAAGCTCCCTGTCTCCAACACCTGTCCGGACCGATTGTACC<br>GCATACTTCGGTACTTGGTTCACGTTCGCCTTCTCAGAGTGG<br>CAGAGGTCGACGGCTTGAAAAAGTACTTGCTCGCTCCTGCTG<br>CGAAATTGCTACTTAGGAACACTGAGAAGAGCATGGTACCCA<br>TCATTCTAGGCATGACCCAAAAGGATTTTGTGGTGCCATGGC<br>ATCACATAAACGATGGTTTGGGGAGTGAAGGTGCCACCGCCT<br>TCGATAAGTCCATGGGAATGTCCTTCTTTGAGTACTTAGAAGA<br>GAATCCTAGCCAGAGCAAGCTCTTCAATGAAGCCATGGCTGG<br>CGAGACTAGGCTTTTAACAGCTTCTCTTATCAATGGTTGCAAA<br>GACTTGTTTCAAGGTATTGAGTCTTTGGTGGATGTTGGAGGA<br>GGGAATGGCACAACCATTAAGGCCATATCTGATTCTTTTCCTC<br>ATATCAAGTGTACCCTCTTCGACCTACCGTACGTTGTTGCAG<br>ATTCTCACGACGACCCTAATATCAAGCGCGTCCATGGTGACA<br>TGTTCAAGTCCATCCCTAGTTCCCAAGCCATCTTGCTCAAGTT<br>GATTTTGCATGATTGGAGTGACGAAGATTGTGTGAAGATTCTA<br>AAGCGATGCAAGGAAGCTGTGCCTAAGGAAGGAGGGAAGGT<br>GATAATAGTAGATGTAGCACTGGATGAGGAGTCTCAGCATGA<br>GTTGAGTAGCACAAGATTGATACTTGACATAGATATGTTGGTC<br>AACACTGGAGGCAAGGAGAGGAGCAAAGAGGACTGGGAGAA<br>GCTTATCAAATGTGCAGGATTTGGAGGGTACAAAATTAGGCA<br>CATTGCTGCTATTCAGTCAGTCATAGAGGTCTTCCCATAGCA<br>GTAGTAGTGGTGATTAATAACTGTGTAGAGTGATCTTGTGTTC<br>GTGAGAAACTCTGCTTGATCTGTTGTCGTCTAATAAAGCTCTC<br>ATTTTCATCTGTTTCCAGTTTTCTAGAAAGCTGTTAATTAATAT<br>TATCCTACCTGTTTTTCTATGCTTCATTATTGCTTAACTGCG

Supplementary Table 3 | Strains and plasmids used in this study.

Strain	Description	Source
BL21 (DE3)	*fhuA2 [lon] ompT gal (λ DE3) [dcm] ΔhsdS*	New England BioLabs

Plasmid	Description	Source
pHis8-4b-His_8-SaDAH	His_8-SaDAH (T7), *lacI*, *lacO*, Kan^R, F1-ori	This study
pHis8-4b-His_8-McDAH	His_8-McDAH (T7), *lacI*, *lacO*, Kan^R, F1-ori	This study
pHis8-4b-His_8-SaDAH G226D	His_8-SaDAH G226D (T7), *lacI*, *lacO*, Kan^R, F1-ori	This study

Supplementary Table 4 | Oligonucleotide sequences reported in this study.

**Name**	**Sequence (5′ - 3′)**
His_8-SaDAH-F	GAAAACTTGTACTTCCAGGCCCATGGCATGGAAGTCGAGAAGGTACTCC
His_8-SaDAH-R	CGGGCTTTGTTAGCAGCCGGATCGCCATGGCTATTTTGAGGTCACTTTCGCTAC
His_8-McDAH-F	GAAAACTTGTACTTCCAGGCCCATGGAAGTTGAAAAAGTCCTCCAGCCGATTTCC
His_8-McDAH-R	CGGGCTTTGTTAGCAGCCGGATCGCCATGGCTATTTAGATGTGACTTTCGCTAC
His_8-SaDAH G226D-F (QuikChange)	GTGTAGTCCCTCATACAGACTATCCTGCAATGACAAT
His_8-SaDAH G226D-R (QuikChange)	ATTGTCATTGCAGGATAGTCTGTATGAGGGACTACAC

Supplementary References

1. Edgar, R. C. MUSCLE: multiple sequence alignment with high accuracy and high throughput. *Nucleic Acids Res.* **32**, 1792–1797 (2004).
2. Robert, X. & Gouet, P. Deciphering key features in protein structures with the new ENDscript server. *Nucleic Acids Research* vol. 42 W320–W324 (2014).
3. Babiker, H. A. *et al.* Biosynthetic Relationship between Acutumine and Dechloroacutumine in Menispermum dauricum Root Cultures. *Biosci. Biotechnol. Biochem.* **63**, 515–518 (1999).
4. Mitchell, A. J. *et al.* Structural basis for halogenation by iron- and 2-oxo-glutarate-dependent enzyme WelO5. *Nat. Chem. Biol.* **12**, 636–640 (2016).
5. Shan, J. *et al.* Liquid Chromatography Coupled with Linear Ion Trap Hybrid OrbitrapMass Spectrometry for Determination of Alkaloids in. *Molecules* **23**, (2018).
6. King, S. M., Calandra, N. A. & Herzon, S. B. Total syntheses of (–)-acutumine and (–)-dechloroacutumine. *Angew. Chem. Int. Ed Engl.* **52**, 3642–3645 (2013).
